# Antiproliferative benzothiazoles incorporating a trimethoxyphenyl scaffold as novel colchicine site tubulin polymerisation inhibitors

**DOI:** 10.1080/14756366.2020.1753721

**Published:** 2020-04-17

**Authors:** Dong-Jun Fu, Si-Meng Liu, Fu-Hao Li, Jia-Jia Yang, Jun Li

**Affiliations:** a Modern Research Center for Traditional Chinese Medicine, School of Chinese Materia Medica, Beijing University of Chinese Medicine, Beijing, People’s Republic of China; b Department of Gastroenterology, The Fifth Affiliated Hospital of Zhengzhou University, Zhengzhou, People’s Republic of China; c Department of Pharmacy, People’s Hospital of Zhengzhou, Zhengzhou, People’s Republic of China

**Keywords:** Colchicine site tubulin polymerisation inhibitor, prostate cancer, benzothiazole, bioisosterism

## Abstract

Tubulin polymerisation inhibitors exhibited an important role in the treatment of patients with prostate cancer. Herein, we reported the medicinal chemistry efforts leading to a new series of benzothiazoles by a bioisosterism approach. Biological testing revealed that compound **12a** could significantly inhibit *in vitro* tubulin polymerisation of a concentration dependent manner, with an IC_50_ value of 2.87 μM. Immunofluorescence and EBI competition assay investigated that compound **12a** effectively inhibited tubulin polymerisation and directly bound to the colchicine-binding site of β-tubulin in PC3 cells. Docking analysis showed that **12a** formed hydrogen bonds with residues Tyr357, Ala247 and Val353 of tubulin. Importantly, it displayed the promising antiproliferative ability against C42B, LNCAP, 22RV1 and PC3 cells with IC_50_ values of 2.81 μM, 4.31 μM, 2.13 μM and 2.04 μM, respectively. In summary, compound **12a** was a novel colchicine site tubulin polymerisation inhibitor with potential to treat prostate cancer.

## Graphical abstract

**Figure:**
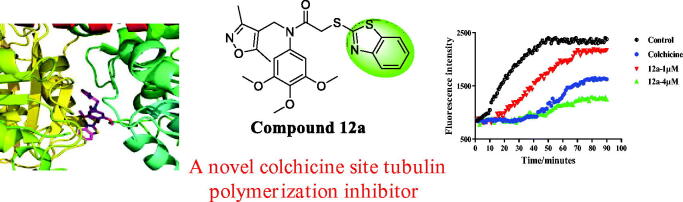

## Introduction

Prostate cancer is the second most commonly diagnosed cancer and the sixth leading cause of cancer death among men worldwide, with an estimated 1276000 new cancer cases and 359000 deaths in 2018^1–3^. Microtubules play an important role in various cellular processes, including spindle formation, cellular shape maintenance, and intracellular transportation^4–6^. Microtubule has been an attractive target for chemotherapeutic agents to treat prostate cancer ^7^. Anticancer agents targeting tubulin bind at four binding sites: taxanes, vinca alkaloids, colchicine and laulimalide sites^8^^,^^9^. Although some tubulin inhibitors that bind to vinca alkaloids and taxanes sites have been approved by Food and Drug Administration (FDA), there are no FDA approved colchicine site tubulin inhibitors currently. Therefore, it is necessary to develop novel tubulin polymerisation inhibitors targeting the colchicine binding site to treat prostate cancer.

**Figure 1.**
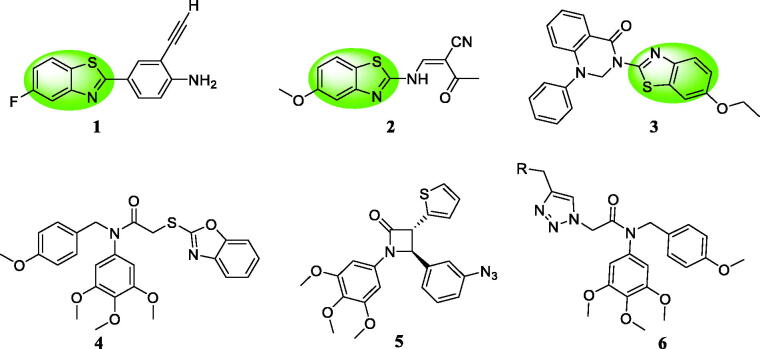
Benzothiazole analogues **1**∼**3** and colchicine site tubulin inhibitors **4**∼**6** as anticancer agents.

Recently, benzothiazole scaffold has been an attractive subject due to its potently anticancer activity in medicinal chemistry^10–12^. 2–(4-Aminophenyl) benzothiazole analogue **1** (Figure 1) exhibited the antiproliferative activity against MCF-7 and MDA-468 cells^13^^,^^14^. Benzothiazole **2** induced apoptosis and inhibited the growth of HL60 and U937 cells^15^. Benzothiazole linked a phenylpyridopyrimidinone **3** showed the significant cytotoxicity against human cervical cancer cell line ME-180 with an IC_50_ value of 4.01 μM^16^. Based on these findings, benzothiazole was a promising scaffold to design anticancer agents. On the other hand, our group also reported three series of colchicine site tubulin inhibitors: (1) compound **4** could change the membrane potential of the mitochondria against MGC-803 cells^17^; (2) β-lactam containing a 3,4,5-trimethoxyphenyl unit **5** exhibited the well antiproliferative activity against MGC-803 cells *in vitro* and *in vivo*^18^; (3) compound **6** could inhibit MGC803 cell growth and colony formation, induce G2/M phase arrest and promote apoptosis^19^.

**Figure 2.**
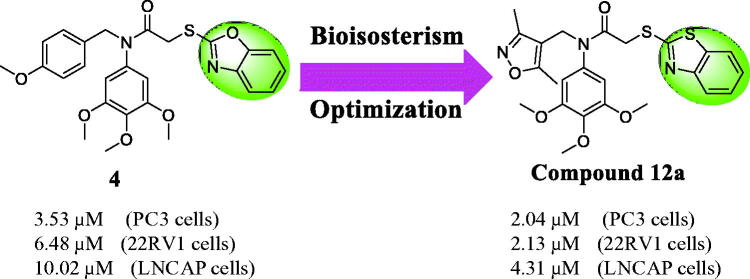
The rational design of benzothiazole analogues by bioisosterism approach.

Bioisosterism is a useful strategy in rational drug design to improve pharmacodynamic or pharmacokinetic properties of lead compounds ^20^. In order to optimise the reported colchicine site tubulin inhibitor **4** and discovery more potently anticancer agents^21–25^, benzothiazole was used as a bioisostere of the benzoxazole in the lead structure. Herein, we synthesised a variety of benzothiazole derivatives based on the lead compound **4** by the bioisosterism approach. In addition, the preliminary structure activity relationship (SAR) was explored. The rational design of benzothiazole analogues was described in Figure 2. Compared with the reported compound **4**, compound **12a** showed the better antiproliferative activity against prostate cancer cells.

## Materials and methods

### Chemistry

#### General procedure for preparation of compounds 9a∼9k and 12a∼12d

Imine intermediates **7a∼7k** and **10a∼10d** were synthesised according to our reported references^17^,^19^. A solution of intermediates **7a∼7k** and **10a∼10d** (3 mmol) in dichloromethane (20 ml) was reacted with 2-chloroacetyl chloride (6 mmol) at room temperature. After the mixture was stirred for 3 h, the reaction mixture was diluted with water. The organic layer was washed by aqueous NaHCO_3_, water, and brine, dried over anhydrous Na_2_SO_4_. The system was concentrated to provide the crude products **8a∼8k** and **11a∼11d** without purification.

Benzo[*d*]thiazole-2-thiol (1 mmol) and sodium hydroxide (2 mmol) were added to the solution of crude products **8a∼8k** and **11a∼11d** (1 mmol) in acetone (5 ml). After stirring at 60 °C for 5 h, the reaction mixture was concentrated to remove acetone. Then, the system was treated with a solution of ethyl acetate (30 ml) and H_2_O (30 ml). The organic layer was washed with aqueous Na_2_CO_3_ solution, water, and brine. The organic system was concentrated to obtain the residue, which was purified by column chromatography with *n*-hexane/ethyl acetate (10:1) to afford the target compounds **9a∼9k** and **12a∼12d**.

#### 2-(Benzo[d]thiazol-2-ylthio)-N-(4-fluorobenzyl)-N-(3,4,5-trimethoxyphenyl)acetamide (9a)

Yield: 52%, white solid, m.p:1 4 3 ∼ 144 °C. ^1^H NMR (400 MHz, CDCl_3_) δ 7.67 (d, *J* = 7.9 Hz, 1H), 7.60 (d, *J* = 8.1 Hz, 1H), 7.37–7.29 (m, 1H), 7.24 – 7.21 (m, 1H), 7.15 (dd, *J* = 8.5, 5.5 Hz, 2H), 6.87 (t, *J* = 8.7 Hz, 2H), 6.27 (s, 2H), 4.79 (s, 2H), 3.98 (s, 2H), 3.75 (s, 3H), 3.65 (s, 6H). ^13 ^C NMR (100 MHz, CDCl_3_) δ 166.04, 164.53, 152.70, 137.06, 135.82, 134.54, 132.04, 129.95, 129.87, 124.95, 123.37, 120.18, 114.33, 114.12, 104.75, 104.72, 59.91, 55.18, 51.88, 35.49. HRMS (ESI+): *m/z* [M + H]^+^ calcd for C_25_H_24_FN_2_O_4_S_2_: 499.1162; found: 499.1167.

#### 2-(Benzo[d]thiazol-2-ylthio)-N-(3-chlorobenzyl)-N-(3,4,5-trimethoxyphenyl)acetamide (9 b)

Yield: 69%, white solid, m.p:1 0 5 ∼ 106 °C. ^1^H NMR (400 MHz, CDCl_3_) δ 7.72–7.53 (m, 2H), 7.34–7.27 (m, 1H), 7.24–7.20 (m, 1H), 7.17 (d, *J* = 1.4 Hz, 2H), 7.12 (dd, *J* = 9.6, 5.3 Hz, 2H), 6.29 (s, 2H), 4.79 (s, 2H), 4.00 (s, 2H), 3.76 (s, 3H), 3.66 (s, 6H). ^13 ^C NMR (100 MHz, CDCl_3_) δ 166.18, 164.45, 152.77, 151.80, 138.14, 137.18, 135.77, 134.54, 133.17, 128.76, 128.17, 126.81, 126.37, 124.98, 123.37, 120.29, 120.10, 104.73, 59.92, 55.21, 52.12, 35.47. HRMS (ESI+): *m/z* [M + H]^+^ calcd for C_25_H_24_ClN_2_O_4_S_2_: 515.0866; found: 515.0870.

#### 2-(Benzo[d]thiazol-2-ylthio)-N-(4-chlorobenzyl)-N-(3,4,5-trimethoxyphenyl)acetamide (9c)

Yield: 27%, white solid, m.p:1 0 7 ∼ 109 °C. ^1^H NMR (400 MHz, CDCl_3_) δ 7.67 (d, *J* = 7.9 Hz, 1H), 7.57 (d, *J* = 8.0 Hz, 1H), 7.37–7.29 (m, 1H), 7.23 (dd, *J* = 11.1, 4.1 Hz, 1H), 7.19 – 7.05 (m, 4H), 6.29 (s, 2H), 4.79 (s, 2H), 3.98 (s, 2H), 3.75 (s, 3H), 3.66 (s, 6H). ^13 ^C NMR (100 MHz, CDCl_3_) δ 166.13, 164.48, 152.74, 151.77, 137.10, 135.84, 134.70, 134.55, 132.50, 129.58, 127.56, 124.98, 123.39, 120.18, 120.14, 104.70, 59.92, 55.22, 52.02, 35.42. HRMS (ESI+): *m/z* [M + H]^+^ calcd for C_25_H_24_ClN_2_O_4_S_2_: 515.0866; found: 515.0869.

#### 2-(Benzo[d]thiazol-2-ylthio)-N-(4-bromobenzyl)-N-(3,4,5-trimethoxyphenyl)acetamide (9d)

Yield: 82%, white solid, m.p:1 3 0 ∼ 131 °C. ^1^H NMR (400 MHz, CDCl_3_) δ 7.70–7.63 (m, 1H), 7.56 (d, *J* = 7.8 Hz, 1H), 7.37–7.28 (m, 3H), 7.25–7.20 (m, 1H), 7.07 (d, *J* = 8.4 Hz, 2H), 6.29 (s, 2H), 4.77 (s, 2H), 3.97 (s, 2H), 3.75 (s, 3H), 3.65 (s, 6H). ^13 ^C NMR (100 MHz, DMSO-d_6_) δ 161.86, 160.43, 148.52, 147.34, 132.89, 131.59, 130.97, 130.21, 126.30, 125.71, 120.82, 119.23, 116.40, 115.92, 100.48, 55.70, 51.01, 47.87, 31.26. HRMS (ESI+): *m/z* [M + H]^+^ calcd for C_25_H_24_BrN_2_O_4_S_2_: 559.0361; found: 559.0368.

#### 2-(Benzo[d]thiazol-2-ylthio)-N-(4-methylbenzyl)-N-(3,4,5-trimethoxyphenyl)acetamide (9e)

Yield: 43%, white solid, m.p:1 4 4 ∼ 146 °C. ^1^H NMR (400 MHz, CDCl_3_) δ 7.64 (dd, *J* = 16.8, 8.0 Hz, 2H), 7.33–7.28 (m, 1H), 7.26–7.20 (m, 1H), 7.06 (d, *J* = 8.0 Hz, 2H), 6.99 (d, *J* = 7.9 Hz, 2H), 6.26 (s, 2H), 4.78 (s, 2H), 4.00 (s, 2H), 3.75 (s, 3H), 3.63 (s, 6H), 2.25 (s, 3H). ^13 ^C NMR (100 MHz, CDCl_3_) δ 165.94, 164.76, 152.62, 151.75, 137.02, 136.23, 135.97, 134.49, 133.12, 128.19, 128.04, 124.93, 123.35, 120.30, 120.07, 104.89, 59.90, 55.17, 52.37, 35.70, 20.11. HRMS (ESI+): *m/z* [M + H]^+^ calcd for C_26_H_27_N_2_O_4_S_2_: 495.1412; found: 495.1417.

#### 2-(Benzo[d]thiazol-2-ylthio)-N-benzyl-N-(3,4,5-trimethoxyphenyl)acetamide (9f)

Yield: 69%, white solid, m.p:1 5 0 ∼ 152 °C. ^1^H NMR (400 MHz, CDCl_3_) δ 7.65 (dd, *J* = 14.2, 8.0 Hz, 2H), 7.34–7.27 (m, 1H), 7.24–7.15 (m, 6H), 6.25 (s, 2H), 4.83 (s, 2H), 4.00 (s, 2H), 3.74 (s, 3H), 3.62 (s, 6H). ^13 ^C NMR (100 MHz, CDCl_3_) δ 166.00, 164.62, 152.62, 151.85, 136.99, 136.18, 135.89, 134.55, 128.19, 127.42, 126.59, 124.93, 123.33, 120.28, 120.09, 104.80, 59.90, 55.15, 52.58, 35.62. HRMS (ESI+): *m/z* [M + H]^+^ calcd for C_26_H_27_N_2_O_5_S_2_: 511.1361; found: 511.1367.

#### 2-(Benzo[d]thiazol-2-ylthio)-N-(3-methoxybenzyl)-N-(3,4,5-trimethoxyphenyl)acetamide (9 g)

Yield: 69%, white solid, m.p:1 2 3 ∼ 124 °C. ^1^H NMR (400 MHz, CDCl_3_) δ 7.64 (dd, *J* = 14.7, 8.0 Hz, 2H), 7.36–7.26 (m, 1H), 7.24–7.19 (m, 1H), 7.10 (t, *J* = 8.1 Hz, 1H), 6.74 (dd, *J* = 6.0, 3.2 Hz, 3H), 6.28 (s, 2H), 4.80 (s, 2H), 4.01 (s, 2H), 3.75 (s, 3H), 3.63 (d, *J* = 7.2 Hz, 9H). ^13 ^C NMR (100 MHz, CDCl_3_) δ 166.02, 164.57, 158.68, 152.63, 151.85, 137.68, 137.04, 135.93, 134.52, 128.36, 124.95, 123.34, 120.51, 120.32, 120.05, 113.49, 112.38, 104.82, 59.90, 55.18, 54.17, 52.58, 35.6. HRMS (ESI+): *m/z* [M + H]^+^ calcd for C_26_H_27_N_2_O_5_S_2_: 511.1361; found: 511.1367.

#### 2-(Benzo[d]thiazol-2-ylthio)-N-(4-methoxybenzyl)-N-(3,4,5-trimethoxyphenyl)acetamide (9 h)

Yield: 52%, white solid, m.p:1 2 5 ∼ 126 °C. ^1^H NMR (400 MHz, CDCl_3_) δ 7.63 (dd, *J* = 18.4, 7.9 Hz, 2H), 7.39–7.25 (m, 1H), 7.24–7.15 (m, 1H), 7.09 (d, *J* = 8.6 Hz, 2H), 6.71 (d, *J* = 8.6 Hz, 2H), 6.25 (s, 2H), 4.76 (s, 2H), 3.98 (s, 2H), 3.75 (s, 3H), 3.70 (s, 3H), 3.64 (s, 6H). ^13 ^C NMR (100 MHz, CDCl_3_) δ 165.83, 164.65, 158.08, 152.60, 151.81, 136.93, 135.93, 134.52, 129.57, 128.35, 124.89, 123.31, 120.27, 120.08, 112.70, 104.83, 59.90, 55.17, 54.24, 51.99, 35.66. HRMS (ESI+): *m/z* [M + H]^+^ calcd for C_26_H_27_N_2_O_5_S_2_: 511.1361; found: 511.1366.

#### 2-(Benzo[d]thiazol-2-ylthio)-N-(4-methoxybenzyl)-N-(p-tolyl)acetamide (9i)

Yield: 78%, yellow liquid. ^1^H NMR (400 MHz, CDCl_3_) δ 7.66 (t, *J* = 8.1 Hz, 2H), 7.31 (t, *J* = 7.7 Hz, 1H), 7.21–7.17 (m, 1H), 7.05 (dd, *J* = 8.2, 3.6 Hz, 4H), 6.91 (d, *J* = 8.1 Hz, 2H), 6.69 (d, *J* = 8.4 Hz, 2H), 4.76 (s, 2H), 3.92 (d, *J* = 1.0 Hz, 2H), 3.70 (s, 3H), 2.23 (s, 3H). ^13 ^C NMR (100 MHz, CDCl_3_) δ 165.97, 157.93, 137.75, 137.45, 129.33, 129.29, 128.20, 127.22, 124.89, 123.26, 120.39, 120.00, 112.70, 54.18, 52.12, 35.97, 20.07. HRMS (ESI+): *m/z* [M + H]^+^ calcd for C_24_H_23_N_2_O_2_S_2_: 435.1201; found: 435.1207.

#### 2-(Benzo[d]thiazol-2-ylthio)-N-(3,4-dichlorophenyl)-N-(4-methoxybenzyl)acetamide (9j)

Yield: 63%, yellow liquid. ^1^H NMR (400 MHz, CDCl_3_) δ 7.66 (t, *J* = 6.1 Hz, 2H), 7.37–7.29 (m, 2H), 7.27–7.20 (m, 2H), 7.02 (d, *J* = 8.4 Hz, 2H), 6.89 (dd, *J* = 8.4, 2.0 Hz, 1H), 6.69 (d, *J* = 8.4 Hz, 2H), 4.76 (s, 2H), 3.94 (s, 2H), 3.71 (s, 3H). ^13 ^C NMR (100 MHz, CDCl_3_) δ 165.73, 164.19, 158.19, 151.46, 139.70, 134.46, 132.48, 131.99, 130.24, 129.64, 129.22, 127.34, 127.22, 125.03, 123.44, 120.37, 120.10, 112.94, 54.21, 52.13, 35.30. HRMS (ESI+): *m/z* [M + H]^+^ calcd for C_23_H_19_Cl_2_N_2_O_2_S_2_: 489.0265; found: 489.0269.

#### 2-(Benzo[d]thiazol-2-ylthio)-N-(4-methoxybenzyl)-N-phenylacetamide (9k)

Yield: 68%, white solid, m.p: 109–110 °C. ^1^H NMR (400 MHz, CDCl_3_) δ 7.66 (t, *J* = 8.6 Hz, 2H), 7.29 (dt, *J* = 15.1, 7.7 Hz, 4H), 7.21 (d, *J* = 7.6 Hz, 1H), 7.09–7.01 (m, 4H), 6.68 (d, *J* = 8.6 Hz, 2H), 4.79 (s, 2H), 3.92 (s, 2H), 3.69 (s, 3H). ^13 ^C NMR (100 MHz, CDCl_3_) δ 165.80, 164.67, 157.97, 151.79, 140.41, 134.50, 129.28, 128.76, 128.05, 127.52, 124.87, 123.26, 120.43, 120.02, 112.73, 54.18, 52.13, 36.01. HRMS (ESI+): *m/z* [M + H]^+^ calcd for C_23_H_21_N_2_O_2_S_2_: 421.1044; found: 421.1049.

#### 2-(Benzo[d]thiazol-2-ylthio)-N-((3,5-dimethylisoxazol-4-yl)methyl)-N-(3,4,5-trimethoxyphenyl)acetamide (12a)

Yield: 83%, white solid, m.p:1 3 7 ∼ 138 °C. ^1^H NMR (400 MHz, CDCl_3_) δ 7.67 (d, *J* = 7.9 Hz, 1H), 7.57 (d, *J* = 8.1 Hz, 1H), 7.39–7.28 (m, 1H), 7.26–7.21 (m, 1H), 6.31 (s, 2H), 4.65 (s, 2H), 3.92 (s, 2H), 3.76 (s, 3H), 3.69 (s, 6H), 1.95 (s, 3H), 1.90 (s, 3H). ^13 ^C NMR (100 MHz, CDCl_3_) δ 167.43, 165.70, 164.36, 158.95, 153.02, 151.69, 137.51, 134.80, 134.47, 125.14, 123.46, 120.16, 120.05, 109.21, 105.09, 60.05, 55.39, 39.84, 35.19, 9.74, 8.91. HRMS (ESI+): *m/z* [M + H]^+^ calcd for C_24_H_26_N_3_O_5_S_2_: 500.1314; found: 500.1317.

#### 2-(Benzo[d]thiazol-2-ylthio)-N-(pyridin-4-ylmethyl)-N-(3,4,5-trimethoxyphenyl)acetamide (12 b)

Yield: 65%, white solid, m.p:1 2 9 ∼ 130 °C. ^1^H NMR (400 MHz, CDCl_3_) δ 8.43 (d, *J* = 5.5 Hz, 2H), 7.69 (d, *J* = 7.7 Hz, 1H), 7.63 (d, *J* = 8.1 Hz, 1H), 7.38–7.31 (m, 1H), 7.25 (d, *J* = 7.2 Hz, 1H), 7.20 (dd, *J* = 6.5, 4.5 Hz, 2H), 6.39 (s, 2H), 4.85 (s, 2H), 4.02 (s, 2H), 3.75 (s, 3H), 3.67 (s, 6H). ^13 ^C NMR (100 MHz, CDCl_3_) δ 166.59, 164.51, 152.92, 151.71, 148.28, 145.96, 137.29, 135.91, 134.61, 125.13, 123.54, 122.71, 120.31, 119.97, 104.41, 59.92, 55.27, 52.00, 35.15. HRMS (ESI+): *m/z* [M + H]^+^ calcd for C_24_H_24_N_3_O_4_S_2_: 482.1208; found: 482.1213.

#### 2-(Benzo[d]thiazol-2-ylthio)-N-((6-chloropyridin-3-yl)methyl)-N-(3,4,5-trimethoxyphenyl)acetamide (12c)

Yield: 73%, white solid, m.p:1 4 5 ∼ 146 °C. ^1^H NMR (400 MHz, CDCl_3_) δ 8.13 (d, *J* = 2.2 Hz, 1H), 7.73–7.60 (m, 2H), 7.52 (d, *J* = 8.1 Hz, 1H), 7.38–7.30 (m, 1H), 7.23 (dd, *J* = 11.3, 4.0 Hz, 1H), 7.12 (d, *J* = 8.2 Hz, 1H), 6.34 (s, 2H), 4.80 (s, 2H), 3.96 (s, 2H), 3.76 (s, 3H), 3.69 (s, 6H). ^13 ^C NMR (100 MHz, CDCl_3_) δ 166.47, 164.30, 153.00, 151.66, 149.88, 148.93, 138.94, 137.36, 135.60, 134.53, 130.76, 125.06, 123.47, 123.28, 120.20, 120.05, 104.57, 59.94, 55.31, 49.59, 35.13. HRMS (ESI+): *m/z* [M + H]^+^ calcd for C_24_H_23_ClN_3_O_4_S_2_: 516.0819; found: 516.0826.

#### 2-(Benzo[d]thiazol-2-ylthio)-N-(naphthalen-2-ylmethyl)-N-(3,4,5-trimethoxyphenyl)acetamide (12d)

Yield: 52%, white solid, m.p:1 1 8 ∼ 119 °C. ^1^H NMR (400 MHz, CDCl_3_) δ 7.76–7.71 (m, 1H), 7.65 (dt, *J* = 10.7, 5.0 Hz, 3H), 7.55 (d, *J* = 8.5 Hz, 2H), 7.42–7.33 (m, 3H), 7.24–7.17 (m, 2H), 6.26 (s, 2H), 4.99 (s, 2H), 4.02 (s, 2H), 3.73 (s, 3H), 3.54 (s, 6H). ^13 ^C NMR (100 MHz, CDCl_3_) δ 166.13, 164.56, 152.63, 151.81, 137.00, 135.89, 134.53, 133.61, 132.16, 131.79, 127.21, 127.19, 126.79, 126.58, 126.12, 125.12, 124.97, 124.91, 123.32, 120.29, 120.07, 104.81, 59.88, 55.11, 52.75, 35.63. HRMS (ESI+): *m/z* [M + H]^+^ calcd for C_29_H_27_N_2_O_4_S_2_: 531.1412; found: 531.1415.

## Biology

### Cell culture and MTT assay

PC3, C42B, 22RV1, and LNCAP cell lines were cultured in an atmosphere containing 5% CO_2_ at 37 °C, with RPMI-1640 medium with 10% foetal bovine serum, 100 U/ml penicillin and 0.1 mg/ml streptomycin. Cells were seeded at a density of 1500 per well in 96-well plates for 72 h. Then, 20 μL MTT (thiazolyl blue tetrazolium bromide) solution was added to each well, and incubated for 4 h at 37 °C. 150 μL DMSO was added to each well to dissolve the formazan after removing the liquid, the absorbance was determined at 570 nm.

### *In vitro* tubulin polymerisation assay

Tubulin (5.6 mg/ml) was resuspended in PEM buffer (containing 80 mM PIPES, 1 mM ATP, 1 mM EGTA, 10.2% glycerol, 0.5 mM MgCl_2_) and then was preincubated with compound **12a**, colchicine or vehicle DMSO on ice. The reaction was monitored by a spectrophotometer in absorbance at 420 nm (excitation wavelength is 340 nm).

### Immunostaining and microscopy

PC3 cells were seeded on the slices and incubated overnight. Then, cells were treated with different concentrations of **12a**. After 48 h, slices were fixed by 4% paraformaldehyde for 15 min after washed by PBS for 3 times. 0.5% Triton-X-100 was added and shaked for 20 min. 0.1% BSA was used to block for 30 min and then removed. The slices were added β-tubulin antibody (1:100) and incubated overnight. Then slices were washed by PBST 3 times, bind with secondary antibody with FITC signal (1:500) in a dark. DAPI was used to stain for 3 min and then removed. After that, images were captured by Laser scanning confocal microscope (Nikon, Japan).

### EBI competition assay

6-Well plates were seeded with PC3 cells for 24 h. Then, cells were incubated with compound **12a**, colchicine or DMSO for 2 h and afterward treated with EBI (100 mM) for 2 h. Then, the cells were harvested and lysed. Cell extracts were used for Western blotting analysis.

### Western blot analysis

Cellular protein lysates were denatured in loading buffer at 100 °C prior to adding into SDS-PAGE. Proteins were transferred to nitrocellulose membranes, probed with indicated antibodies, and visualised by an enhanced chemiluminescence detection system. The western blotting bands were semi-quantified using Image J and adjusted for loading control.

### DAPI staining

Cells were seeded on a 6-well plate (2 × 105/well) and treated with the test compound after the incubation for 24 h. Then, cells were stained by DAPI (4′,6-diamidino-2-phenylindole) in the dark for 30 min. The images of cells were observed in a fluorescence microscope (Nikon, Japan).

### Molecular modelling

Molecular modelling was performed using the autodock 4 software from the Scripps Research Institute. Molecules were first generated using Pymol software. For the receptor preparation, the PDB code (1SA0) was downloaded from the Protein Data Bank. Dockings were initially performed using a blind docking method on a lower resolution docking. This was followed by a series of resolving steps to attempt to fine tune the compound. The final results were analysed by Pymol software.

## Results and discussion

### Synthesis of the target analogues 9a∼9k and 12a∼12d

**Scheme 1.**
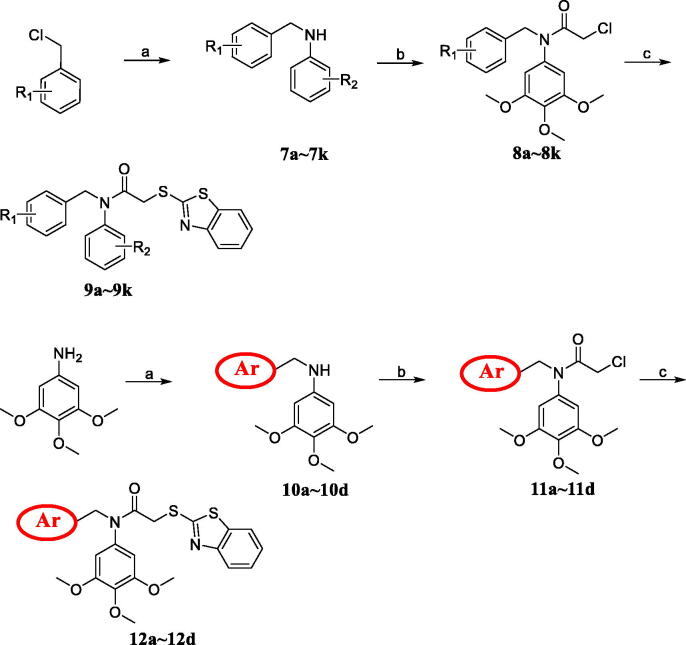
Reagents and conditions: (a) K_2_CO_3_, aniline derivatives or various benzyl chlorides, acetone, reflux; (b) Chloroacetyl chloride, dichloromethane, rt; (c) NaOH, benzo[d]thiazole-2-thiol, acetone, reflux.

The synthetic route of benzothiazoles containing the 3, 4, 5-trimethoxyphenyl fragment was shown in Scheme 1. Aniline derivatives and various benzyl chlorides were subjected to nucleophilic substitution reaction to afford imines **7a∼7k** and **10a∼10d** in the presence of potassium carbonate. The imine intermediates were reacted with chloroacetyl chloride to obtain the amide analogues **8a∼8k** and **11a∼11d** in the dichloromethane system. The target analogues **9a∼9k** and **12a∼12d** were easily obtained at the reflux condition with benzo[d]thiazole-2-thiol in the presence of sodium hydroxide. The chemical structures of all benzothiazole derivatives **9a∼9k** and **12a∼12d** were characterised using spectral methods, and all spectral data corroborated the structures.

### Antiproliferative activity of novel benzothiazole derivatives

To discovery novel anti-prostate cancer agents and tubulin polymerisation inhibitors, we evaluated the antiproliferative activity of all benzothiazole derivatives **9a∼9k** and **12a∼12d** against a prostate cancer cell line 22RV1 using the 3–(4,5-dimethyl-2-thiazolyl)-2,5-diphenyl-2-*H*-tetrazolium bromide (MTT) assay. Based on previous references, 5-fluorouracil (5-Fu) and was usually selected as a reference drug in the MTT assay to evaluate the antiproliferative activity of synthetic benzothiazole derivatives^26^^,^^27^. Therefore, 5-Fu was used as the control drug in this work. In addition, compound **4** was also the control drug to compare the antiproliferative activity.

From the antiproliferative results of benzothiazole derivatives in Table 1, benzothiazoles without the 3, 4, 5-trimethoxyphenyl fragment (**9i∼9k**) displayed very weak activity against 22RV1 cells with IC_50_ values of >20 μM. However, benzothiazole-trimethoxyphenyl hybrids **9a∼9h** showed moderate to potent activity with IC_50_ values from 5.24 μM to 19.07 μM against 22RV1 cells. Based on this findings, it revealed that 3, 4, 5-trimethoxyphenyl fragment exhibited an important role for the antiproliferative activity of these benzothiazole derivatives.

**Table 1.** Antiproliferative activity of the synthetic derivatives **9a∼9k** against 22RV1 cells.


Compound	R_1_	R_2_	**22RV1**^a^
**9a**	4-F	3,4,5-triOCH_3_	16.23 ± 0.84
**9b**	3-Cl	3,4,5-triOCH_3_	19.07 ± 1.15
**9c**	4-Cl	3,4,5-triOCH_3_	12.17 ± 0.58
**9d**	4-Br	3,4,5-triOCH_3_	6.20 ± 0.71
**9e**	4-CH_3_	3,4,5-triOCH_3_	8.25 ± 0.43
**9f**	H	3,4,5-triOCH_3_	9.06 ± 0.17
**9g**	3-OCH_3_	3,4,5-triOCH_3_	7.37 ± 0.56
**9h**	4-OCH_3_	3,4,5-triOCH_3_	5.24 ± 0.78
**9i**	4-OCH_3_	4-CH_3_	>20
**9j**	4-OCH_3_	3,4-diCl	>20
**9k**	4-OCH_3_	H	>20
**4**	–	–	6.48 ± 0.25
**5-FU**	–	–	18.31 ± 1.27

^a^ Antiproliferative activity was assayed by exposure for 72 h.

To obtain potently antiproliferative compounds, trimethoxyphenyl fragment as B ring was reserved and the molecular deversity of different substituent groups attaching on the A ring was explored. Among them, compound **9 h** showed the most potent activity with an IC_50_ value of 5.24 μM against 22RV1 cells. We found that the substitution on the A ring was important for the activity showing an over 3-fold activity loss, when the *p*-Br group was replaced with the *m*-Cl group (compounds **9d** *vs*. **9 b**). In addition, compounds **9 g∼9h** with an electron-donating methoxy group on the A ring had a more potent inhibitory effect (7.37 and 5.24 μM, respectively) against 22RV1 cells than compounds **9 b∼9c** (IC_50_ >12 μM) with an electron-withdrawing group. From the biological data of compounds **9a∼9h**, we can conclude that the substituents on the A ring had a remarkable effect on their cytotoxic activity.

To determine whether the aromatic ring or heterocycles have an effect on the activity, compounds with a 3, 5-dimethylisoxazole ring (**12a**), a pyridine ring (**12 b**), a 2-chloropyridine ring (**12c**), and a naphthalene ring (**12d**) were synthesised and their antiproliferative activity results were shown in Table 2. Replacement of the pyridine scaffold of compound **12 b** with a 2-chloropyridine fragment (**12c**) or a naphthalene unit (**12d**) led to a loss of the activity. However, changing the pyridine ring of compound **12 b** to a 3, 5-dimethylisoxazole ring of compound **12a** led to an improvement of the activity against 22RV1 cells. All these results indicated that 3, 5-dimethylisoxazole displayed the better inhibitory activity than other selected heterocycles and phenyl ring.

**Table 2.** Antiproliferative activity of the synthetic derivatives **12a∼12d** against 22RV1 cells.


Compound		22RV1
**12a**		2.13 ± 0.24
**12b**		5.09 ± 0.62
**12c**		11.84 ± 0.91
**12d**		6.93 ± 0.46
**4**	–	6.48 ± 0.25
**5-FU**	–	18.31 ± 1.27

^a^ Antiproliferative activity was assayed by exposure for 72 h.

### Compound 12a inhibited cell proliferation against prostate cancer

**Figure 3.**
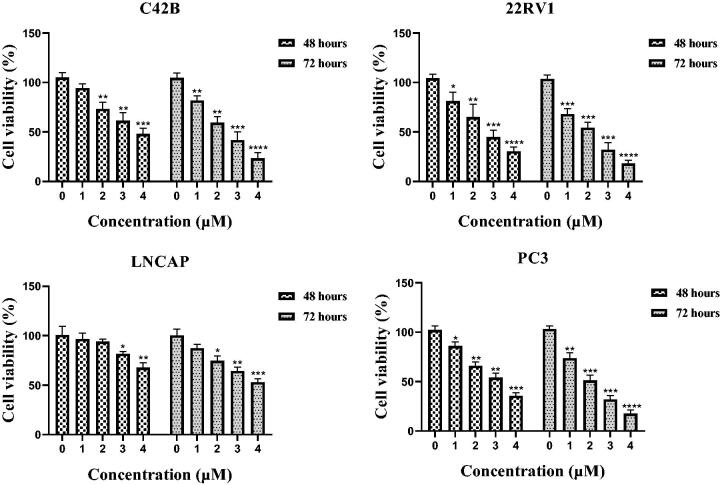
Antiproliferative activity of benzothiazole **12a** against four prostate cancer cell lines for 48 h and 72 h. *p < 0.05 verse control, **p < 0.01 verse control, ***p < 0.001 versus control, ****p < 0.0001 versus control.

Among all benzothiazole derivatives **9a∼9k** and **12a∼12d**, **12a** displayed the most potently antiproliferative activity with an IC_50_ value of 2.13 μM against 22RV1 cells. Compound **12a** was further examined for possible cytotoxicity against RWPE-1 (normal human prostatic cell line). We found that compound **12a** exhibited no cytotoxicity against RWPE-1 (>20 μM). The results indicated that compound **12a** had good selectivity between the selected cancer cell line (22RV1) and a normal cell line (RWPE-1). To evaluate its antiproliferative activity against other prostate cancer cell lines, C42B, LNCAP and PC3 cells were selected to perform MTT assay. As shown in the Figure 3, benzothiazole derivative **12a** exhibited the well antiproliferative activity against C42B, LNCAP and PC3 cell lines with IC_50_ values of 2.81 ± 0.43 μM, 4.31 ± 0.27 μM and 2.04 ± 0.51 μM, respectively.

### Compound 12a inhibited tubulin polymerisation by immunostaining assay

**Figure 4.**
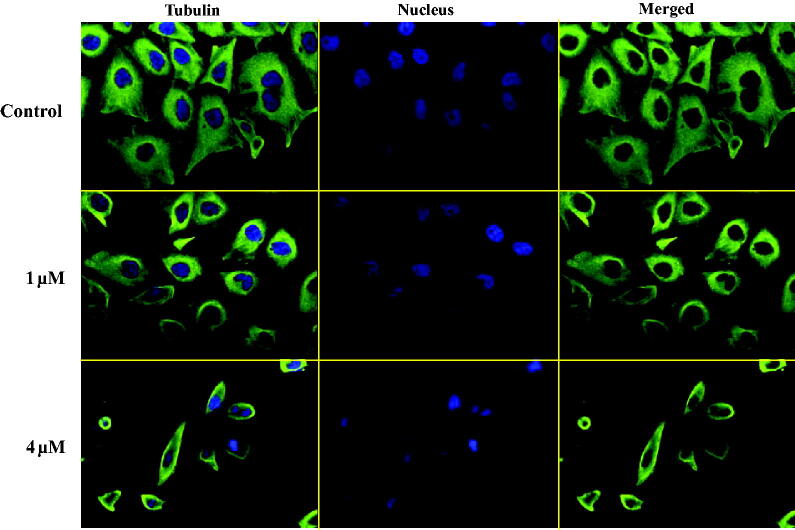
Compound **12a** showed strong depolymerising effects on the interphase microtubule network.

As the microtubule system played an important role in the maintenance of cell shape and basic cellular functions, an immunofluorescence staining assay was performed to study whether benzothiazole hybrid **12a** could disrupt the microtubule dynamics in living cells^28^. As shown in Figure 4, in the control group, the microtubule network in PC3 cells exhibited normal arrangement and organisation, characterised by regularly assembled, normal microtubules wrapped around the cell nucleus. Benzothiazole hybrid **12a** at the concentration of 1 μM moderately depolymerised interphase microtubules whereas **12a** at the higher concentration of 4 μM induced much stronger depolymerisation effects. These immunofluorescence experiments indicated that compound **12a** inhibited tubulin polymerisation in a concentration dependent manner against PC3 cells.

### Compound 12a inhibited tubulin polymerisation targeting the colchicine binding site

**Figure 5.**
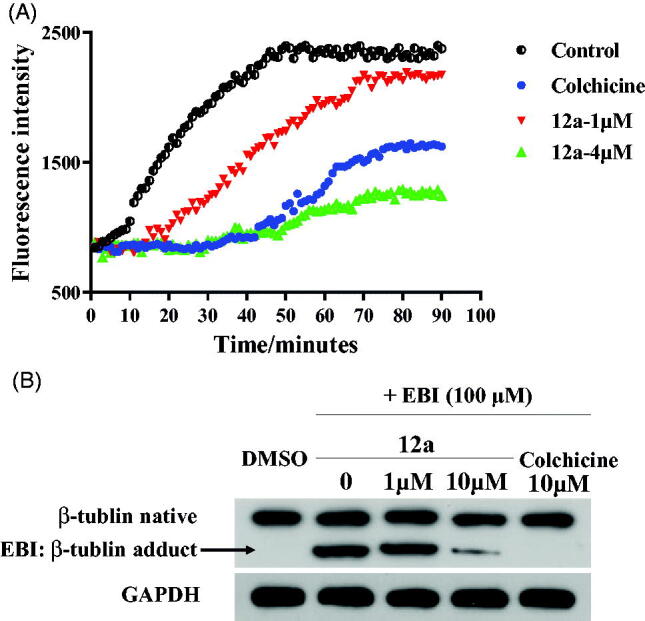
(A) Tubulin polymerisation inhibitory activity of **12a**. (B) EBI competition assay of **12a** in PC3 cells.

The *in vitro* tubulin polymerisation inhibition activity of compound **12a** was evaluated because of its best antiproliferative activity results among all benzothiazole hybrids. When tubulin was incubated with the tested compound **12a**, the increased tendency of the fluorescence intensity was obviously slowed down compared with the control. The IC_50_ value of compound **12a** was 2.87 μM against tubulin (Figure 5(A)). It revealed thatbenzothiazole hybrid **12a** was a novel tubulin polymerisation inhibitor.

In order to evaluate whether benzothiazole hybrid **12a** directly binds to tubulin at the colchicine binding site, we carried out *N*,*N′*-ethylenebis(iodoacetamide) (EBI) competition assay in PC3 cells according to previously published references^29^^,^^30^. EBI is an alkylating agent that has the property to specifically cross-link the Cys239 and the Cys354 residues of β-tubulin involved in the colchicine-binding site^31^. The occupancy of colchicine-binding site by tubulin polymerisation inhibitors could inhibit the formation of the EBI: β-tubulin adduct. Preincubation of compound **12a** at 10 μM prevented the formation of the EBI: β-tubulin adduct, resulting in the decrease of the adduct band comparing with DMSO and EBI treatment. Thus, the assay (Figure 5(B)) indicated that compound **12a** directly bind to the colchicine-binding site of β-tubulin.

### Compound 12a induced morphological changes and affected the expression of apoptosis-related proteins

**Figure 6.**
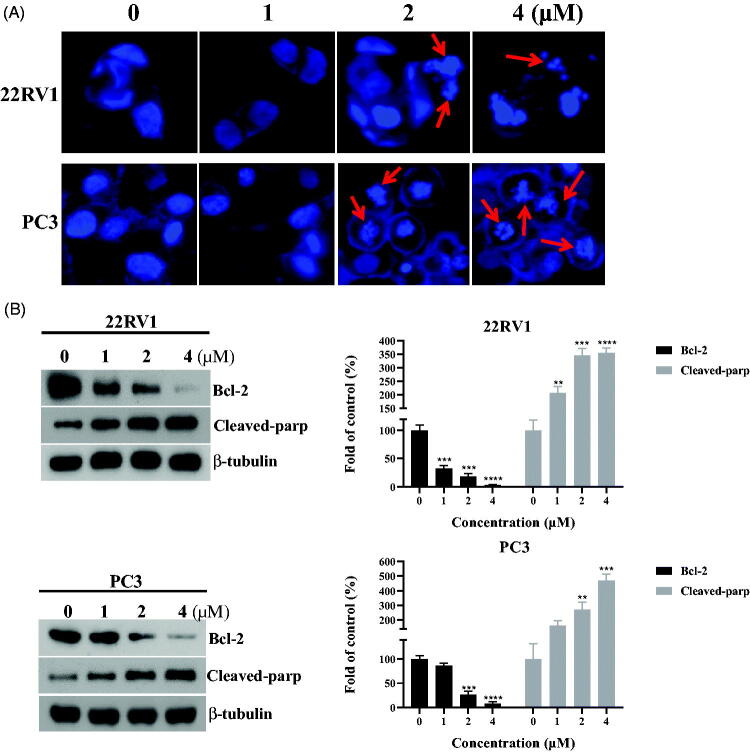
(A) Morphological changes of prostate cancer cells with the treatment of **12a**. (B) The expression levels of apoptosis-related proteins (Bcl-2 and Cleaved-parp). **p < 0.01 versus control, ***p < 0.001 versus control, ****p < 0.0001 versus control.

As most tubulin destabilising agents displayed the ability to induce morphological changes and affect the expression of apoptosis-related proteins^32^, DAPI staining and western blotting experiments were performed to determine the effects of benzothiazole hybrid **12a**. After 48 h incubation with compound **12a** (0, 1 μM, 2 μM and 4 μM), characteristic apoptotic morphological changes were observed by fluorescence microscopy, including chromatin shrinkage and formation of apoptotic bodies (Figure 6(A)). The studies revealed that compound **12a** induced morphological changes of prostate cancer (22RV1 cells and PC3 cells) possibly by inducing apoptosis.

Bcl-2 family proteins are key regulators of apoptosis and overexpression of the prominent pro-survival Bcl-2 family members like Bcl-2 is a common feature responsible for deregulation of apoptosis in prostate cancer cells^33^. In order to investigate the apoptosis effects of compound **12a**, the expression of apoptosis-related proteins (Bcl-2 and Cleaved-parp) were tested. As shown in the Figure 6(B), the expression level of Bcl-2 was decreased and the expression level of Cleaved-parp was increased in a concentration-dependent manner against prostate cancer (22RV1 cells and PC3 cells).

### Molecular modelling analysis of compound 12a

In the current work, trimethoxyphenyl-benzothiazole hybrid **12a** has been identified as a novel colchicine site tubulin polymerisation inhibitor by the immunostaining, *in vitro* tubulin polymerisation assay and EBI competition assay. Furthermore, molecular docking methodologies were also used to explore any molecular interaction exist between 3,4,5-trimethoxyphenyl-benzothiazole hybrid **12a** and residues lies in the active site cativity of tubulin. We have used Autodock as an automated tool to perform docking and selected PDB code 1SA0.

**Figure 7.**
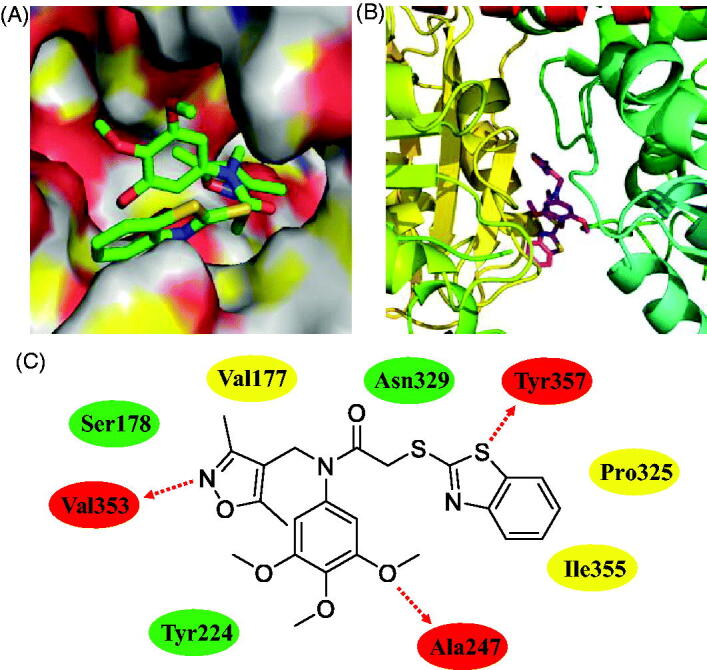
Molecular docking studies of compound **12a**. (A) Compound **12a** bound to β-tubulin (surface). (B) The complex formed between tubulin and compound **12a**. (C) Hydrogen bonds (red ellipse), hydrophilic interactions (green ellipse) and hydrophobic interactions (yellow ellipse) observed between compound **12a** and residues.

As shown in Figure 7, benzo[*d*]thiazole, 3,4,5-trimethoxyphenyl ring, and isoxazole unit formed three hydrogen bonds with residues Tyr357, Ala247, and Val353, respectively. This finding illustrated the importance of 3,4,5-trimethoxyphenyl scaffold for tubulin polymerisation inhibitors. 3,4,5-Trimethoxyphenyl-benzothiazole hybrid **12a** formed hydrophilic interactions with the residues of Ser178, Asn329 and Tyr224. In addition, **12a** also formed hydrophobic interactions with the residues of Pro325, Val177 and Ile355. All these results suggested that hybrid **12a** as a novel tubulin polymerisation inhibitor could display the potently antiproliferative activity.

## Conclusions

A series of benzothiazole derivatives were designed, synthesised and evaluated for their antiproliferative activity against prostate cancer. Among them, compound **12a** possessed the potently antiproliferative ability against C42B, LNCAP, 22RV1 and PC3 cell lines with IC_50_ values of 2.81 μM, 4.31 μM, 2.13 μM and 2.04 μM, respectively. Molecular mechanisms illustrated that compound **12a** could induce morphological changes and affect the expression levels of apoptosis-related proteins (Bcl-2 and Cleaved-parp) against prostate cancer cells. From the *in vitro* tubulin polymerisation inhibition assay and EBI competition assay, compound **12a** was a novel colchicine site tubulin polymerisation inhibitor. Taken together, compound **12a** might be a lead candidate for its further development in treatment of prostate cancer.
